# The effects of transcranial direct current stimulation on attention and inhibitory control of children and adolescents with attention-deficit/hyperactivity disorder (ADHD): Study protocol for a randomized, sham-controlled, triple-blind, cross-over trial

**DOI:** 10.1097/MD.0000000000024283

**Published:** 2021-02-26

**Authors:** Rachel Silvany Quadros Guimarães, Igor D. Bandeira, Bianca Lima Barretto, Thiago Lima Barretto, Thamires Wanke, Clara Oliveira Carvalho Alves, Chrissie Ferreira de Carvalho, Pedro H. Lucena, Luciana Rodrigues-Silva, Rita Lucena

**Affiliations:** a Postgraduate Program in Medicine and Health, Medical School of Bahia; b Laboratory of Neuropsychopharmacology, Psychiatry Division of the Professor Edgard Santos University Hospital; c Medical School of Bahia; d Institute of Psychology, Federal University of Bahia, Salvador; e Department of Psychology, Federal University of Santa Catarina, Florianópolis; f Bahiana School of Medicine and Public Health; g Department of Pediatrics; h Department of Neuroscience and Mental Health, Medical School of Bahia, Federal University of Bahia, Salvador, Brazil.

**Keywords:** attention-deficit/hyperactivity disorder, neuromodulation, noninvasive brain stimulation, prefrontal cortex, transcranial direct current stimulation

## Abstract

Attention-deficit/hyperactivity disorder (ADHD) is characterized by a persistent pattern of inattention and hyperactivity/impulsivity. Despite the proven efficacy of pharmacological treatment, many individuals continue to suffer socially and academically and some experience significant side effects that negate the use psychotropic drugs. Transcranial direct current stimulation (tDCS) is a cortical neuromodulation feature that has shown positive results in the treatment of various neuropsychiatric conditions.

**Objectives:**

To investigate the effect of tDCS on the performance of children and adolescents with ADHD in the neuropsychological tests of visual attention, verbal, and inhibitory control.

**Methodology:**

Triple blind, randomized, sham-controlled, cross-over trial involving tDCS in children and adolescents with ADHD. Initial screening will be performed using Swanson, Nolan, and Pelham – IVand Wechsler intelligence scale for children fourth edition vocabulary and cube subtests. Individuals will be evaluated pre-tDCS and post-tDCS with the Wechsler intelligence scale for children fourth edition Digitus subtest, neuropsychological assessment battery second edition inhibiting responses subtest, Corsi cubes, and visual attention test-4.

## 1 Introduction

Attention-deficit/hyperactivity disorder (ADHD) is a prevalent developmental disorder characterized by inadequate levels of attention, hyperactivity and impulsivity and frequently comorbid with other psychiatric disorders.^[1]^ According to the American Academy of Pediatrics, different areas of the child's life may be compromised as a result of ADHD, such as academic performance, interpersonal relationships and well-being, raising the need for effective treatments.^[1,2]^ The main current therapeutic strategies recommended and widely used are based in psychostimulants and behavioral therapies. They have proven efficacious and cost-effective in the short term,^[1]^ however, there have been reports in the literature of adverse effects in children and adolescents with the use of some of these medications, among them tachyphylaxis, increased blood pressure and heart rate, insomnia, and reduced appetite.^[3–6]^

Some studies report a relationship between ADHD symptoms and executive function deficits.^[7]^ The central executive functions are cognitive flexibility, inhibitory control (self-control and self-regulation), and working memory. The most complex executive functions include problem solving, reasoning, and planning.^[8]^ Inhibitory control, the ability to inhibit prepotent actions, is altered in children and adults with ADHD.^[9]^ It has been suggested that repetitive transcranial magnetic stimulation and transcranial direct current stimulation (tDCS) on the dorsolateral prefrontal cortex could improve inhibitory control, impulsivity, and decision-making.^[10]^

In the past decades, several interventions and diagnostic tools have been tested for brain and mind disorders.^[11–23]^ Among them, tDCS is an emerging neuromodulation technique that uses low-intensity electrical stimulation to modulate target brain regions and has a potential therapeutic benefit because the neurophysiological effect of current applied is durable over time after the stimulation ceases.^[24]^ The anodic tDCS is based on an electric current from a generator that aims to stimulate certain cortical areas, causing an increase in local cerebral blood flow.^[25]^ This tool has been currently used in the treatment of various psychiatric and neurological disorders such as schizophrenia,^[26]^ dyslexia,^[27]^ autism,^[28]^ cerebellar ataxia,^[29]^ epilepsy,^[30]^ unipolar and bipolar depression,^[31]^ since it is considered safe and relatively less expensive than other noninvasive brain stimulation approaches.^[32,33]^ Advantages of the cost-effectiveness of tDCS could be even more important considering low-middle income countries.^[34,35]^

Previous studies have shown that tDCS is both a safe and an encouraging option in the treatment of ADHD,^[10,36–40]^ proving the validity of researching this method and its adverse effects. In addition, some children and adolescents present contraindications for the use of psychoactive drugs, necessitating the use of alternative methods. The pilot open-label trial conducted by Bandeira et al^[38]^ showed improvement in some executive functions such as selective attention and inhibitory control using tDCS in children and adolescents with ADHD. However, further studies with appropriate randomization, blinding, and larger sample sizes should be performed to further investigate these findings. The main objective of this study is to widen the scope of the previous investigation and to prove reproducibility of the findings as well as ensure safety of the technique in this population.

## 2 Study aims

### 2.1 Principal

To determine the impact of tDCS on attention and inhibitory control in children and adolescents with ADHD.

### 2.2 Secondary

To identify the adverse effects related to tDCS in children and adolescents with ADHD.

## 3 Material and methods

### 3.1 Study design

This is a randomized, crossover, triple-blind, sham-controlled study. The study was registered on December 28th, 2017 with Brazilian Registry of Clinical Trials (ReBec) as “The Effects of Transcranial Direct Current Stimulation on Attention and Inhibitory Control of Children and Adolescents with Attention-Deficit/Hyperactivity Disorder (ADHD): a randomized, sham-controlled, triple blind, cross-over trial” (http://www.ensaiosclinicos.gov.br/rg/RBR-7h5qzf/).

### 3.2 Participants and enrollment

#### 3.2.1 Casuistry

The sample will consist of 15 individuals of both genders from the Attention Deficit Hyperactivity Disorder Outpatient Clinic of the Professor Edgard Santos University Hospital, Federal University of Bahia without pharmacological treatment during tDCS intervention.

#### 3.2.2 Definitive diagnostic criteria

The diagnosis of ADHD will be performed by neuropediatricians based on the criteria of the 5th edition of the diagnostic and statistical manual of mental disorders (DSM-5).

### 3.3 Eligibility criteria

#### 3.3.1 Inclusion criteria

(1) Individuals aged between 6 and 16;
(2) Diagnosed with ADHD according to DSM-5 criteria;
(3) Absence of psychiatric comorbidities;
(4) Right handed;
(5) Literate and enrolled in regular schools;
(6) Living in Salvador (Brazil) or metropolitan area;
(7) No pharmacological treatment during tDCS intervention;
(8) Electroencephalogram without epileptogenic activity;
(9) Consent of those responsible for participation in the study.

#### 3.3.2 Exclusion criteria:

(1) Sensory deficit.
(2) Not conforming to any of the above criteria

### 3.4 Sample calculation

The sample size calculation was performed based on a pilot study. We used the variable “errors by default” of visual attention test (TAVIS) test as the primary outcome. Considering the level of significance of 5%, the power of 80%, the mean difference between paired groups of 1.2 and the standard deviation of 1.24, the sample size calculation resulted in 11 subjects. Assuming 25% of dropout, our final sample size was 14 subjects.

### 3.5 Randomization

The individuals participating in the study will be allocated to the sham group, which will not receive effective stimulation, and to the active group, in which tDCS will actually be performed. The allocation will be made randomly by an individual not involved with the study using the instrument available on randomization.com. A list with the allocation will be sealed in an opaque envelope, retained by one of the study authors and only opened on the first day of stimulation, by the same individual. Only the researchers who apply tDCS will have access to this information. After the defined interval of 1-month the individuals who underwent tDCS will be moved to the sham group and vice-versa.

### 3.6 Blinding

The tDCS devices will be covered during the procedure and no patient or relative will have contact with them. To certify blinding of participants in the sham group in relation to the sensation of current flow involved in the procedure, the device will be switched on for 1 minute at the beginning of the stimulation, turned off for the subsequent 28 minutes and reconnected at the last minute of stimulation. The family, the research subject, the evaluators, and the statistician will be blind to the group to which the subject belongs (“Sham” or “Active”). At the end of the 5 tDCS or sham-tDCS sessions in the first stage of the study, patients and their families will be asked if they believed they were in the intervention or control group. After the 30 day washout period, the second stage of the study will be carried out in the same manner, with the same questioning at the end of the 5 sessions of tDCS or sham-tDCS.

### 3.7 Variables

(1) Sex: 0-female; 1-male; 9-not declared;
(2) Age, in years and months;
(3) Grade level;
(4) School: 0-public; 1-private;
(5) Age of diagnosis of ADHD;
(6) Use of medication to treat ADHD: 0-yes; 1-no;
(7) Repeated school years;
(8) Age of school inclusion;
(9) Monitoring by neuropediatrician: 0-yes; 1-no;
(10) Education of the responsible adult: 0-no schooling; 1-elementary incomplete; 2- elementary completed; 3- high-school incomplete, 4-high-school completed; 5-college incomplete; 6-college completed; 7-postgraduate;
(11) Intelligence Quotient;
(12) Average basic reaction time to the TAVIS instrument;
(13) Raw number of errors by omission using TAVIS;
(14) Raw number of hits using TAVIS;
(15) Raw score of vocabulary subtest with Wechsler abbreviated intelligence scale;
(16) Raw score of forward order of digits with Wechsler intelligence scale for children fourth edition (WISC-IV);
(17) Raw score of backward order of digits with WISC-IV;
(18) Forward order hits using Corsi block;
(19) Backward order hits using Corsi block;
(20) Total errors with the inhibition subtest.

### 3.8 Interventions

After selecting the sample according to the aforementioned criteria and neuropediatric evaluation, children diagnosed with ADHD will participate in a neuropsychological assessment in which the intellectual level estimation and its performance in the processes of attention, operational memory, and inhibitory control will be investigated. The Swanson, Nolan, and Pelham – IV questionnaire assessing the diagnostic criteria for ADHD from the DSM-5 will be applied to the parents.

A total of 15 individuals will be selected, randomly assigned in 2 groups, 8 of whom were initially in the tDCS group and 7 in the sham group. It will be made clear to those responsible and to the relevant child that tDCS may not be applied. During the stimulation, the same conditions will be reproduced in both groups, except for active treatment in the sham group. The patient will be informed about the procedure before the beginning of the study, with the device being used for a first perception of the current flow for a few seconds.

The tDCS consists of an application of a direct electric current of low amplitude (2 mA) for 30 minutes from 2 electrodes (5 cm × 7 cm) embedded in saline solution. The anode will be positioned in the left anterior dorsal prefrontal cortex (F3 according to the electroencephalogram system 10–20) and the cathode in the supraorbital region on the opposite side. The device used will be the Striat (Ibramed, Amparo-SP, Brazil), approved by the Brazilian National Health Agency (ANVISA). During the stimulation period participants will participate in playful activities involving memory and attention, such as the “Super Lynx” and “Genius” games. They will be carried out by an appropriately trained individual, in 5 sessions, 1 per day, and in the presence of a physician trained to deal with possible intercurrences. Participants may withdraw from participating in the study at any time according to the informed consent form.

After 1 month, the groups will be crossed. Thus the 8 children initially stimulated will be part of the sham group in the second phase and the children initially in the sham group will then receive active stimulation. Before and after the new cycle of stimulation, neuropsychological evaluations will be carried out by applying the aforementioned tests. The family, the research subject, the evaluators, and the statistician will be blind to the subject's group.

At the end of each session, participants and their families will be asked about the presence of the most prevalent adverse effects related to tDCS reported in previous studies.^[36,38,41]^ Any adverse event identified as severe by the attending physicians will be a criteria for exclusion from the study.

The participants will be contacted by telephone the day before the intervention to confirm their participation, thus avoiding possible absences. In order to promote participant retention and complete follow-up, it will be collected outcome data from participants who discontinue or deviate from intervention protocols. In addition, to promote data quality, the data values will be checked by 2 external individuals to avoid incorrect analysis.

### 3.9 Assessment and measures

#### 3.9.1 Neuropsychological evaluation

All participants will be evaluated by neuropsychological tests at 4 different moments: before the first cycle, with an intellectual screening and additional behavioral analysis; after the first cycle and before and after the second cycle. The tests will be applied by neuropsychologists with experience in the evaluation process and using the following instruments:

Characteristics of the sample:

- Intellectual screening – Wechsler abbreviated intelligence scale^[42,43]^: for obtaining the estimated intelligence quotient 2 subtests will be used: the vocabulary subtest that evaluates semantic knowledge and the matrix ratio subtest that evaluates nonverbal logical reasoning ability, a task that evaluate fluid intelligence.
- Child behavior checklist^[44,45]^: this is a questionnaire that assesses social competence and behavioral problems in individuals aged 4 to 18, based on information provided by those responsible.
- Neuropsychological tests to measure results:
- TAVIS-4^[46]^: assesses children aged 6 to 17. The child presses and holds a button on a joystick when it sees a target on a screen. There are 2 versions: ages 7 to 11 (target stimulus – 6 minutes) and ages 12 to 17 (10 minutes). Each task scores: reaction time, commission errors, omission errors, and number of hits. “Commission Errors” records an answer when it should not be given; Error by omission is the lack of response to a target stimulus; Average reaction time (in milliseconds) is the time the child takes to push the button from when the stimulus appears on the screen. Task 1 (selective attention) involves pressing a button when the target stimulus appears. Task 2 (alternating attention) alternates between 2 types of answers to identify identical geometric shapes of the same color. Task 3 assesses sustained attention (concentration) through a continuous performance test.
- Digit span subtest of the WISC-IV^[42]^: is an attention and working memory measure applied to forward (auditory attention) and backward (working memory) digit movement. The examiner reads aloud a sequence of numbers. The child repeats the numbers in the same order in which they were spoken (forward) and then repeats the numbers in reverse order (backward).
- Corsi block^[47]^: evaluate visual working memory. Subject repeats sequences of touches in different cubes. In the forward order, visual attention is tested and in backward order visuospatial sketch of working memory is tested.
- Inhibition is a subtest of attention and executive functions from the developmental neuropsychological assessment battery second edition (NEPSY II)^[48,49]^: assesses ability to inhibit desire to engage in a pleasant task and/or either stop an automatic behavior or alternate between stop and automatic behavior. The examinee looks at a series of stimuli shapes/arrows and must name the shape or direction or give an alternative response, depending on the stimulus color. Errors occur when an incorrect answer is given or is skipped or not corrected. Anything unanswered due to lack of time is considered an incorrected error. Self-corrected errors occur when an incorrect answer is then corrected. Total of errors is the sum of uncorrected errors and self-corrected errors for each condition (naming: select information; inhibition: ability to inhibit an automatic response; switching: ability to switch attention).
- At the end of the research the results will be disclosed to those responsible via a report written by the relevant researcher.

### 3.10 Statistical analyses

To test the hypotheses of the study various inferential statistical tests will be performed with a comparison of means and medians (Student *t* test and Wilcoxon) using the SPSS statistical package for Windows. In the inferences, a significant result will be considered if the probability of type I error is ≤0.05 (5%).

### 3.11 Ethics

The current project was submitted and approved by the Research Ethics Committee of the Medical School of Bahia, Federal University of Bahia in accordance with consent n° 2.351.121 of October 26th, 2017, on the understanding that:

(1) The research project does not include invasive procedures that expose participants to situations of risk;
(2) Only children and adolescents who sign the consent form for minors and whose parents authorize their participation by duly signing the free and informed consent form will be included;
(3) The researchers undertake to keep the child's identification confidential and use the data strictly for academic purposes, namely scientific discussions and the publication of scientific articles;

At the end of the data collection families will be informed about the results obtained in the neuropsychological tests and they will also be informed of the study's final results. Refusal to participate in the study or interruption of participation during the same will not affect any ongoing relationship with the child in respect of the University healthcare services.

## 4 Discussion

The use of tDCS has already been described in the literature as a method of ADHD treatment with some controversial results. In a 2015 study conducted by Soltaninejad et al,^[10]^ 20 adolescents were stimulated with the objective of improving inhibitory control. They were tested during the stimulation process to compare the results between cathodic, anodic, and placebo stimulation. Studies show that the dorsolateral prefrontal cortex is involved with inhibitory control.^[7]^ Given that the left hemisphere is dominant for language in 95% to 99% of right-handed people and 60% to 70% of left-handers,^[50]^ and in order to stimulate the dominant hemisphere of participants, only right-handed individuals were included in the Soltaninejad study, in which the electrodes were positioned in the left dorsolateral prefrontal cortex and right supraorbital region. The intensity of current used in this study was 1.5 mA and the stimulation time was 15 minutes per session, with a 72-hour interval between 3 different sessions. According to the study there were no statistically significant differences between the results of the inhibitory control tests applied in anodic, cathodic, and placebo stimulation.^[10]^

In a 2016 study carried out by Bandeira et al,^[38]^ with 9 children and adolescents, electrodes of the same size and position were used, operating at a current of 2 mA, along with 30 minutes of stimulation for 5 sessions on consecutive days. Neuropsychological tests performed before and after the stimulation showed improvement of some functions such as selective attention and inhibitory control. However, the study was non-randomized and involved a reduced sample, making it necessary to develop a study with a more robust methodology and to enable a more accurate evaluation of the effects of tDCS on attention and inhibitory control and other neurophysiological parameters in a blind, randomized, sham-controlled setting.

In 2016, Breitling et al^[39]^ performed a cross-over study involving 21 patients with ADHD and 21 without the disorder in a control group, with cathodic and anodic stimulation and placebo performed on all patients with at least 1-week interval between them, with 35cm^2^ electrodes and a 1 mA current being used for 20 minutes. An analysis of the results of the neuropsychological tests carried out revealed that there was no significant effect of tDCS between the groups.

Following randomized, double-blind, sham-controlled studies were performed with adolescent dorsolateral prefrontal cortex stimulation, one of which showed improvement of executive function with anodic stimulation and improvement of inhibitory control with cathodic stimulation.^[51]^ Other study showed that anodic stimulation caused a significant reduction of the clinical symptoms of inattention and impulsivity in patients with ADHD compared to the sham-group.^[36]^ Moreover, in a different study anodal stimulation improved motor performance, but worsened accuracy in the working memory paradigm.^[42]^

We hope, with our randomized, cross-over, triple-blind, sham-controlled protocol, to find statistically significant results regarding patterns of attention and inhibitory control identified by the neuropsychological test battery performed.

## Acknowledgments

The authors thank Paulo Mattos and Nayara Argollo for conceding, respectively, the rights to use the TAVIS-4, and NEPSY-II tests. The authors also thank Fernanda Queiros Campbell, Natália Canário, and Anibal Silvany Neto for helping in this clinical trial.

## Author contributions

**Conceptualization:** Rachel Silvany Quadros Guimarães, Igor D. Bandeira, Bianca Lima Barretto, Thiago Lima Barretto, Chrissie Ferreira de Carvalho, Luciana Rodrigues-Silva, Rita Lucena.

**Data curation:** Rachel Silvany Quadros Guimarães, Igor D Bandeira, Bianca Lima Barretto, Thiago Lima Barretto, Thamires Wanke, Clara Oliveira Carvalho Alves.

**Formal analysis:** Rachel Silvany Quadros Guimarães, Igor D. Bandeira, Bianca Lima Barretto, Thiago Lima Barretto, Pedro H. Lucena, Rita Lucena.

**Funding acquisition:** Rita Lucena.

**Investigation:** Rachel Silvany Quadros Guimarães, Igor D. Bandeira, Bianca Lima Barretto, Thiago Lima Barretto, Thamires Wanke, Clara Oliveira Carvalho Alves, Rita Lucena.

**Methodology:** Rachel Silvany Quadros Guimarães, Igor D. Bandeira, Bianca Lima Barretto, Thiago Lima Barretto, Chrissie Ferreira de Carvalho, Pedro H. Lucena, Luciana Rodrigues-Silva, Rita Lucena.

**Project administration:** Rachel Silvany Quadros Guimarães, Igor D. Bandeira, Bianca Lima Barretto, Thiago Lima Barretto, Rita Lucena.

**Resources:** Rachel Silvany Quadros Guimarães, Igor D. Bandeira, Rita Lucena.

**Supervision:** Rachel Silvany Quadros Guimarães, Igor D. Bandeira, Luciana Rodrigues-Silva, Rita Lucena.

**Validation:** Rachel Silvany Quadros Guimarães, Igor D. Bandeira, Bianca Lima Barretto, Chrissie Ferreira de Carvalho, Luciana Rodrigues-Silva, Rita Lucena.

**Visualization:** Rachel Silvany Quadros Guimarães, Igor D. Bandeira, Chrissie Ferreira de Carvalho, Luciana Rodrigues-Silva, Rita Lucena.

**Writing – original draft:** Rachel Silvany Quadros Guimarães, Igor D. Bandeira, Bianca Lima Barretto, Thamires Wanke, Clara Oliveira Carvalho Alves, Chrissie Ferreira de Carvalho, Rita Lucena.

**Writing – review & editing:** Rachel Silvany Quadros Guimarães, Igor D. Bandeira, Bianca Lima Barretto, Thamires Wanke, Clara Oliveira Carvalho Alves, Chrissie Ferreira de Carvalho, Pedro H. Lucena, Luciana Rodrigues-Silva, Rita Lucena.
